# *ATGme*: Open-source web application for rare codon identification and custom DNA sequence optimization

**DOI:** 10.1186/s12859-015-0743-5

**Published:** 2015-09-21

**Authors:** Edward Daniel, Goodluck U. Onwukwe, Rik K. Wierenga, Susan E. Quaggin, Seppo J. Vainio, Mirja Krause

**Affiliations:** Biocenter Oulu, Faculty of Biochemistry and Molecular Medicine, Structural Biochemistry, University of Oulu, Oulu, Finland; Feinberg School of Medicine, Northwestern University, Chicago, IL 60611 USA; Biocenter Oulu, Laboratory of Developmental Biology, InfoTech Oulu, Center for Cell Matrix Research, Faculty of Biochemistry and Molecular Medicine, University of Oulu, Aapistie 5A, FIN-90220 Oulu, Finland

**Keywords:** Codon usage, Sequence optimization, Protein, Translation, DNA

## Abstract

**Background:**

Codon usage plays a crucial role when recombinant proteins are expressed in different organisms. This is especially the case if the codon usage frequency of the organism of origin and the target host organism differ significantly, for example when a human gene is expressed in E. coli. Therefore, to enable or enhance efficient gene expression it is of great importance to identify rare codons in any given DNA sequence and subsequently mutate these to codons which are more frequently used in the expression host.

**Results:**

We describe an open-source web-based application, ATGme, which can in a first step identify rare and highly rare codons from most organisms, and secondly gives the user the possibility to optimize the sequence.

**Conclusions:**

This application provides a simple user-friendly interface utilizing three optimization strategies: 1. one-click optimization, 2. bulk optimization (by codon-type), 3. individualized custom (codon-by-codon) optimization. ATGme is an open-source application which is freely available at: http://atgme.org

## Background

Alternative synonymous codons are not used with equal frequencies in one organism or also when different organisms are compared [1]. Rare codons are those codons which are used with lower frequencies, < 10 ‰, in a specific expression organism such as *Escherichia coli* (*E.coli*) as compared to the original host [2]. Rare codons have for better assessment sometimes been divided into those codons that are used with lower frequencies (5 to 10 ‰) and those used with lowest frequencies (≤5 ‰) [3]). To differentiate between these frequencies, these codons are classified as rare and highly rare codons, respectively [3].

When recombinant gene expression is carried out, codon usage plays a significant role in the efficiency of the host expression system. Gene expression accuracy and efficiency can be reduced if the codon usage frequencies of the organism of origin and the target host organism differ significantly, and rare codons dominate within the sequence [4]. Studies have shown that the presence of rare codons influences gene expression levels [4, 5] and the solubility and amount of the expressed protein [2].

Interestingly, a recent study suggests that some clusters of rare codons (in proteins longer than 300 amino acid residues) called slow-translating regions or slow patches, provide the protein domains enough time to fold accurately [6, 7], and thus playing a role in proper protein folding. In fact, it has been reported that increased rate of translation caused by the elimination of translational pauses due to the rare codons, resulted in improper protein folding and insolubilization [2]. Once the role of such rare codons has been considered codon usage can be optimized prior to protein production to enhance gene expression rates, in any expression system [8]. In this optimization process, rare codons are identified and mutated to more frequently used codons in the host organism without changing the amino acid sequence of the protein. A variety of mathematical and statistical approaches is available to analyze codon usage. These approaches also enable the analysis of codon usage bias in whole groups of organisms and multiple gene sets. This has recently been extensively reviewed [9].

With only a few rare codons present these codons can be changed by point-mutations. However, recent improvements in technology have enabled cost-effective production of synthetic genes, making the simple ordering of an optimized gene sequence a feasible alternative, irrespective of the number of rare codons present in the target gene. Identification of rare codons is done by using a codon usage table of the host organism.

An online database, called “Codon Usage Database” offers access to the codon usage tables of over 35,000 organisms [10, 11]. This database offers the possibility to explore expression of genes in organisms different to the commonly used ones. In contrast to *E.coli*, other organisms can offer post-translational modification systems that might be useful to express mammalian proteins of scientific and industrial interest. The usage frequency values available from the Codon Usage Database represent the mean values of the codon usage based on every gene of a specific organism present in the Genbank® [12] as of June 2007.

Currently, commercially available tools exist which offer researchers the possibility for codon optimization. These applications are usually expensive for small to medium scale laboratories. Additionally, while several openly available codon optimization tools have been created, many of them are no longer available; others require the users to install the software on their computers. Furthermore, they are commonly limited to the application of the codon usage for only a few organisms. Both commercial and non-commercial tools often provide complex results or analysis requiring significant effort or consultation to interpret. Here we describe a simple user-friendly and flexible web-based application, called *ATGme*, which identifies rare codons and gives several options for codon usage optimization.

## Implementation

### Technical details

*ATGme* is an open-source web-based application implemented in HTML/CSS for presentation and pure Javascript for program logic.

### Input and output

**Fig. 1:**
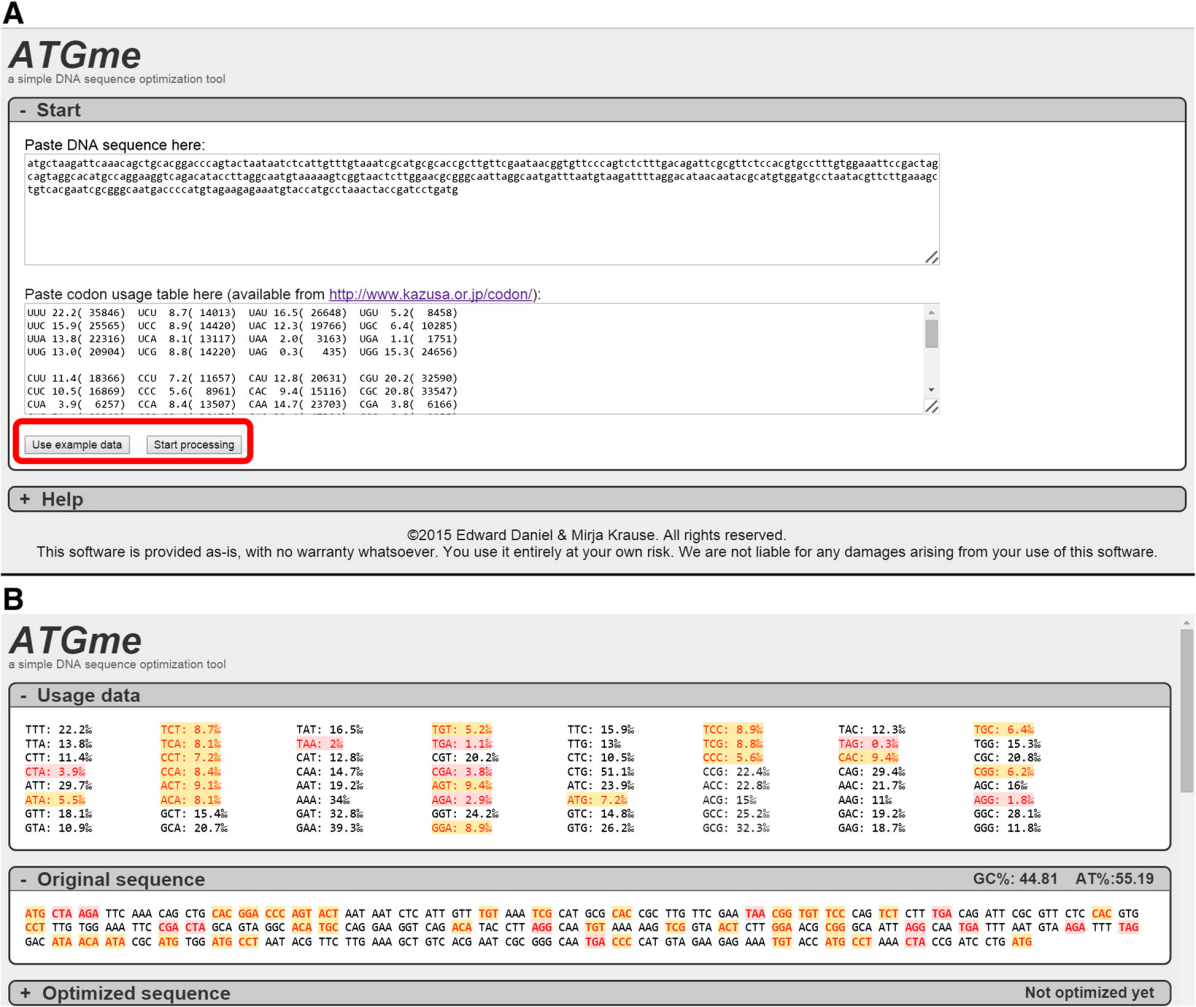
Layout of the web-application. The figure shows the layout of the application. **a** Input window. When “use example data” is clicked (highlighted by a red square) an example sequence and an example codon usage table appears in both Start-boxes. The example input sequence was created as a random sequence with a sequence generator. The example codon table was copied from the codon database from Escherichia coli O157:H7 EDL933. **b** When afterwards the “Start processing” button is clicked (red square) the software highlights all rare codons within the sequence and usage frequencies of the selected host organism are displayed under “usage data”. The sequence can now be optimized

**Fig. 2:**
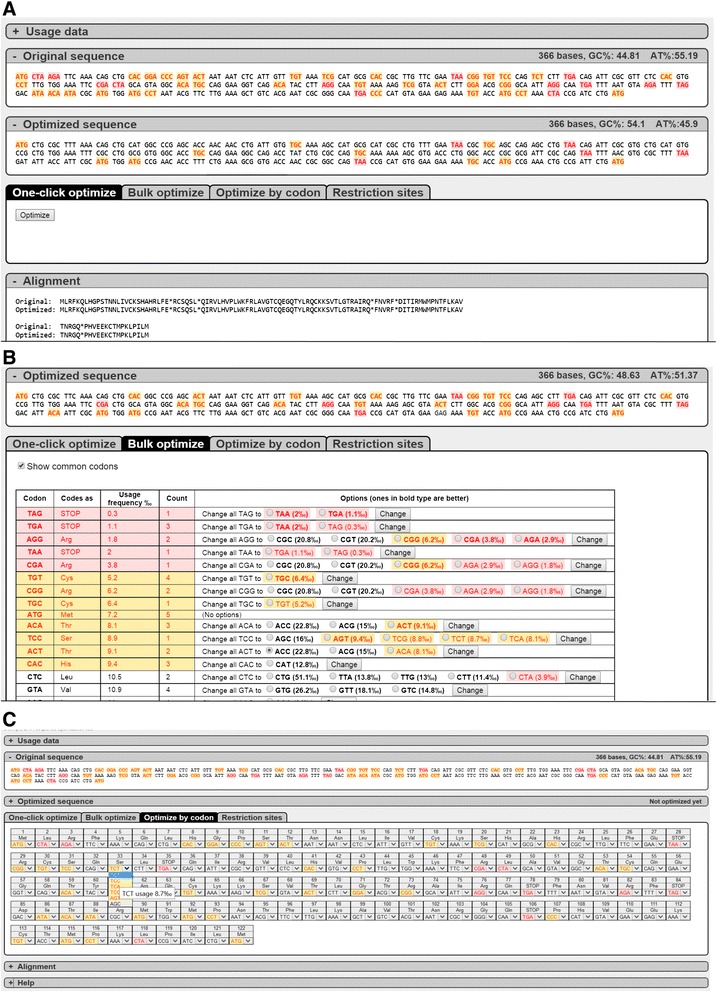
Sequence optimization. This figure shows the example sequence processed with the three possible options to optimize the sequence. **a** shows the “One-click optimize” option. The original sequence with highlighted rare codons can be seen (top) and the optimized sequence is shown (below) with far less rare codons than the original sequence. **b** shows the “bulk-optimization” option. This option can be used to target a certain codon-type. The table listing all the codons of the input sequence provides additional information to the user such as their usage frequency, how often they appear in the target sequence and the possible options to exchange the codon with another one. **c** shows the “optimize by codon” option. Under each codon a drop-down menu shows all the possibilities to exchange a specific individual codon into one that has a higher usage frequency. Hovering over these codons displays their individual usage frequency. All options (**a**-**c**) can also be applied subsequently

The data input requires four steps: (i) Input of the target DNA sequence to be optimized in fasta or text format; (ii) Input of the codon usage table copied from the Codon Usage Database [11] (http://www.kazusa.or.jp/codon/) (Fig. 1). After copying, users can freely modify the usage table according to their needs; (iii) After starting the process, the rare and highly rare codons are highlighted in orange and red respectively in the input sequence as well as in the output sequence (in this step not optimized yet); (iv) The user can then choose between the three different ways to optimize the sequence. a) one-click optimize, b) bulk optimize (per codon-type), c) optimize by codon (individual codon). As the user changes codons the progress is displayed in the automatically updated output sequence. See Fig. 2 for detailed screenshots.

Furthermore, the user has the option to check if his sequence contains certain restriction enzyme recognition sites. Two enzymes can be checked simultaneously. Throughout the process the user can see the alignment of the amino acid sequences of the original and optimized sequences in a separate box (see Fig. 2a). The translation of the DNA sequence into the protein sequence is according to the standard genetic code.

The final output will be the optimized sequence, in which rare codons (if any should remain) would be highlighted in orange and red. Additionally, the A + T and G + C content and the number of bases will be given. To provide an overview throughout the process the usage data is displayed, namely the codon usage table in which rare and highly rare codons are highlighted, respectively.

### Optimization methods

*ATGme* displays rare codons in the target sequence in color. It differentiates between highly rare codons (red) and rare codons (orange). The software offers three different approaches to optimize the target sequence. The first approach (one-click optimize) exchanges all highly rare and rare codons with the most frequently used synonymous counterpart. The second (bulk optimize) exchanges all instances of a specific rare codon always with the same, better (or also worse), codon of the user’s choice. The third approach (optimize by codon) gives the user the possibility to look at the sequence and change each codon one by one. This can be used to address the problem of repetitive elements or also the generation and/or modification of restriction enzyme cleavage sites.

## Results and discussion

Codon optimization for gene design is usually applied to enable and/or increase protein expression levels in a specific host organism. Generally, there are a variety of possible synthetic sequences derived from the starting sequence, which could lead to increased expression levels. How does our software compare to other public web servers and stand-alone applications that allow some kind of codon optimization? Several codon optimization applications have been created over the last decade, but most are not available any longer, like e.g. UpGene [13], GeneDesign [14], GeMS [15], Synthetic Gene Designer [16].

*ATGme* does not need to be downloaded, but is available online free of charge [17]. It will not be at the risk of not being available after some time. In terms of the codon optimization the *ATGme* software applies a highly simplified approach. It will replace rare codons in the target sequence with the single most abundant codon of the host organism of choice (one-click optimize). Additionally, one can have a more detailed look and choose to replace single specific codons with an alternative (not necessarily the most abundant used codon), either all of these codons over the whole sequence (bulk optimize) or one codon at a time (optimize by codon). The latter is especially beneficial to avoid clusters of the same codon throughout the sequence. *ATGme* offers this for any codon usage table present in the Codon Usage Database [11].

As discussed earlier, the usage frequency values the database provides are mean values of the codon usage based on every gene of a specific organism on the GenBank® [11]. In certain cases this is important since e.g. in species under translation selection, the codon usage of highly expressed genes might use a slightly different codon usage than the mean of all genes of a genome. This is commonly known as codon usage bias [18, 19]. In this case it is better to use the codon usage frequency which is calculated for this particular group of highly expressed genes. *ATGme* addresses this topic by the possibility to enter any codon usage table the user would want to employ. There are some applications available which offer sequence optimization, but only for the most commonly used host organisms (e.g. *E.coli*, *Saccharomyces cervisiae*, etc.). OPTIMIZER [20] and JCat [21] offer the longest lists of precomputed codon usage tables. However, the possibilities for the user to customize the codon table input or the optimization result in any way are in most cases not available or very limited. Only services like INKA [22], OPTIMIZER and *ATGme* allow the use of a non-standard genetic code.

In *ATGme* the A + T and G + C content (in %) of the target sequence are calculated from the input and also the output sequence, giving the user the option to influence the ratio by manually choosing suitable codons. Furthermore, the software offers a protein sequence alignment in a separated box (“Alignment”). The given sequences are translated according to standard genetic code, and provide another means of control for the user. The option to address any codon by itself can only be found in two programs available, CodonOpt (*IDT®, Integrated DNA Technolgies*) and *ATGme*.

Both programs show the codon usage frequencies when hovering over each of the possible codons to aid the users in their selection. The latter is additionally simplified by the color-code used in *ATGme*. Furthermore, this function in the *ATGme* software addresses another issue. It is known that imbalanced cellular tRNA pools can lead to frame-shifts during translation [23, 24]. High expression of heterologous proteins can lead to the depletion of certain tRNAs, resulting also in an imbalanced tRNA pool, and finally reduces cell growth [25].

In bacteria and yeast, protein production is regulated by local variations in the translation rate [26]. One such regulation mechanism includes clusters of rare codons which slow down the translation process [26]. Considering this functional role of rare codon clusters, the translational rate of the original organism can be important for a successful overexpression of a heterologous protein. Therefore, a translational rate which resembles the rate in the organism of origin may be beneficial. By addressing each codon separately, the *ATGme* user can choose codons which are not rare, but do not necessarily have the highest usage frequency.

As discussed the introduction of unwanted cleavage sites or ribosome binding sites (RBS) during the optimization process can be a problem. While *ATGme* does not address splicing motifs or RBS, it does however give the user the possibility to check for restriction sites (two enzymes at a time), based on over 100 enzymes which are commercially available and are considered common. In case they should be unwanted, these restriction sites can be addressed (e.g. introduction of silent mutations) with the “Optimize by Codon” option.

## Conclusions

Here we describe a web-based application, called *ATGme*, which identifies rare and highly rare codons displaying them in the input-sequence as colored codons. *ATGme* offers three methods of optimization: 1. one-click optimization, 2. bulk optimization, 3. custom optimization codon-by-codon. Furthermore, the users can identify common restriction sites in their optimized sequences. The software is freely available as an open-source web application [17], and the source code is made available for non-commercial use. Additionally, it gives users the possibility to modify/ optimize the sequence on a codon-by-codon basis to create individualized custom optimized sequences.

## Availability and requirements

**• Project name:** ATGme

**• Project home page:**http://atgme.org [17]

**• Operating system(s):** Platform independent – web-based

**• Programming language:** Javascript

**• Other requirements:** Modern web browser

**• License:** open-source for non-commercial applications

**• Any restrictions to use by non-academics:** license required

## Abbreviations

*E. coli*: *Escherichia coli*
DNA: Deoxyribonucleic acid
RBS: Ribosome binding site
